# Microglia morphophysiological diversity and its implications for the CNS

**DOI:** 10.3389/fimmu.2022.997786

**Published:** 2022-10-19

**Authors:** Andrés Vidal-Itriago, Rowan A. W. Radford, Jason A. Aramideh, Cindy Maurel, Natalie M. Scherer, Emily K. Don, Albert Lee, Roger S. Chung, Manuel B. Graeber, Marco Morsch

**Affiliations:** ^1^ Faculty of Medicine, Health and Human Sciences, Macquarie Medical School, Macquarie University, Sydney, NSW, Australia; ^2^ Brain and Mind Centre, Faculty of Medicine and Health, The University of Sydney, Sydney, NSW, Australia

**Keywords:** microglia, microglial morphology, microglia-neuron interactions, microglia diversity, microglia activation

## Abstract

Microglia are mononuclear phagocytes of mesodermal origin that migrate to the central nervous system (CNS) during the early stages of embryonic development. After colonizing the CNS, they proliferate and remain able to self-renew throughout life, maintaining the number of microglia around 5-12% of the cells in the CNS parenchyma. They are considered to play key roles in development, homeostasis and innate immunity of the CNS. Microglia are exceptionally diverse in their morphological characteristics, actively modifying the shape of their processes and soma in response to different stimuli. This broad morphological spectrum of microglia responses is considered to be closely correlated to their diverse range of functions in health and disease. However, the morphophysiological attributes of microglia, and the structural and functional features of microglia-neuron interactions, remain largely unknown. Here, we assess the current knowledge of the diverse microglial morphologies, with a focus on the correlation between microglial shape and function. We also outline some of the current challenges, opportunities, and future directions that will help us to tackle unanswered questions about microglia, and to continue unravelling the mysteries of microglia, in all its shapes.

## 1 Introduction

**Figure 1:**
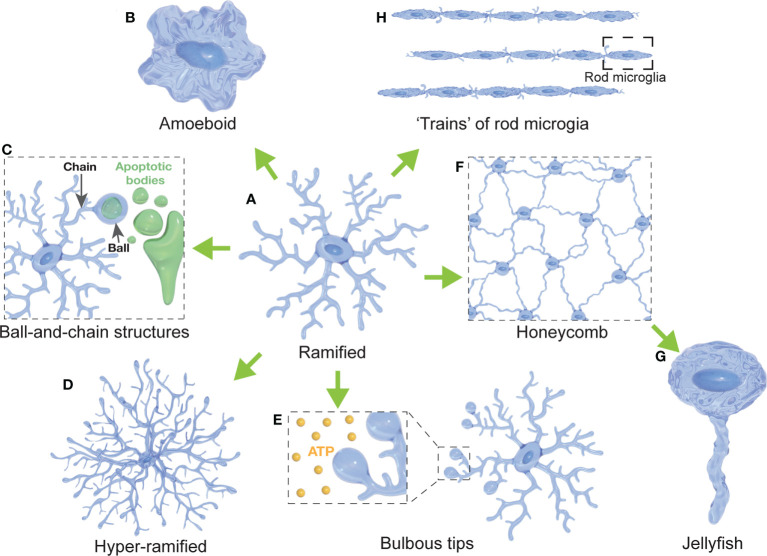
Diversity of microglial morphologies. **(A)** Ramified microglia are highly branched with multiple primary and secondary processes (often considered surveillant). **(B)** Amoeboid microglia present with a highly rounded morphology compared to their ramified states (often with a high phagocytic and migratory capacity). **(C)** Microglia can form ball-and-chain structures at the tip of their processes to phagocytose small amounts of material (such as synapses or apoptotic bodies). **(D)** Hyper-ramified microglia present with increased branching of their processes (often observed in acute and chronic stress models). **(E)** Microglia display bulbous budding at the end of some of their processes (considered to be important for ATP sensing). **(F)** Several microglial cells form a network resembling a honeycomb (reported in response to BBB leakage). **(G)** Jellyfish morphologies have been reported as a morphological transition of honeycomb microglia after extensive astrocytic death in the glia limitans (in response to TBI). **(H)** Rod microglia are characterized by an elongated, narrowed soma without planar processes that can form trains of rod microglial cells (in response to injury).

In the first descriptions of microglia by Pío del Río-Hortega in 1919, microglial activation was depicted as the transition from a ramified morphology (**Figure 1A**) in the ‘resting’ state to an amoeboid morphology (**Figure 1B**) in the’activated’ state (1). Now it is widely accepted that ramified microglia actively screen the CNS, establish contacts with neurons and other cells, and monitor and influence neuronal activity (2–4). However, our understanding of microglial physiology in relation to its morphology is still very limited, and a lack of ramification is generally considered an indicator of microglial activation. New genetic, molecular and pharmacological interventions, combined with novel *in vivo* and *ex vivo* models, have begun to describe new morphological features of microglia physiology and its interactions with other cells, supporting the idea that microglial morphology is highly dynamic and complex (5–7). These studies of microglial morphophysiological heterogeneity provide further evidence that microglia activation cannot simply be reduced to a structural transition from a ramified to an amoeboid shape.

Understanding the relationship between microglial morphology and their precise physiological roles provides critical insights into the spatiotemporal dynamics of microglial responses. In turn, this will lead to a greater understanding of how microglia maintain CNS homeostasis or, on the contrary, contribute to disease etiology. We are now aware of the crucial involvement of microglia in synaptogenesis, synaptic plasticity, axonal regeneration, neuronal survival and regulation of neuronal activity, not only during development but throughout adult life (8–15). These additional functional roles of microglia highlight their importance far beyond their well- known roles in CNS immunity and debris elimination.

This review summarizes the current knowledge regarding the correlation of microglia morphology and function from the early stages of embryonic development throughout the adult life, in health, aging and disease. We highlight some of the specific microglial morphological features that we consider important for their functional spectrum and future classifications. We further discuss the importance of investigating microglial dynamics using *in vivo* and *ex vivo* approaches to better understand the spatiotemporal changes microglia undergo in certain conditions and throughout life.

## 2 Microglial biology

Microglia are myeloid phagocytes and the only innate immune cells permanently residing in the central nervous system (CNS). Microglia comprise between 5% and 12% of all the cells in the murine CNS, with variable densities depending on the CNS region studied (16). Microglia have been historically considered as phagocytes and immune cells that react to neuronal insult and pathological events in the CNS. However, it has become apparent that microglia serve more functions besides phagocytosis and the production of inflammatory cytokines (17, 18). Many studies have provided clear evidence that microglia play crucial physiological roles in the healthy brain; being involved in the development of the CNS connectivity, synaptic plasticity, monitoring of neuronal activity, and maintaining CNS homeostasis into adult life (2, 19–21). To perform such a diverse range of functions, microglia must sense different cues in their immediate microenvironment and adapt their morphology to different stimuli, displaying a plethora of cellular shapes (**Figure 1**). Whether microglia achieve their functional diversity by means of their phenotypic plasticity or through an early diversification into a heterogeneous population of cells is still unknown. The literature states many examples that indicate a correlation between the morphology and function of microglia, i.e. that amoeboid microglia are associated with phagocytosis of cellular debris (2, 22–25). However, the lack of a standard classification of microglia morphophysiology makes it often difficult to compare such associations between studies. It remains unclear whether a certain function is linked to a specific morphology, and/or whether microglia with a certain shape are limited to a particular role.

### 2.1 Microglial ontology

Unlike neurons and other glial cells (oligodendrocytes and astrocytes), microglia are not of neuroectodermal origin. Instead, they are myeloid cells of hematopoietic origin (2). Microglia arise from erythromyeloid precursors generated in the yolk sac, from where they migrate *via* the blood stream to the CNS in the early stages of embryonic development (26–29). After colonizing the CNS, the microglial precursors complete their differentiation and microglia remain a stable CNS-resident population, maintaining their numbers through self-renewal (30, 31), where the signaling of the colony-stimulating factor-1 receptor (CSF1R) is crucial for the proliferation, differentiation and survival of mononuclear phagocytes such as macrophages, osteoclasts and microglia (32). For instance, in the human cortex, microglia have a median age of 4.2 years, replacing 28% of the population every year (33). In the murine cortex, microglia have a median lifetime of more than 15 months with approximately 50% of these cells surviving the entire lifespan of the mouse (34). Some studies have shown that circulating monocytes can enter the CNS during inflammation and pathological incidents, where the blood-brain barrier (BBB) permeability is compromised. However, these cells are classified into a differentiated subset of cells, not contributing to the microglial cell pool and without self-renewal capacity (35–38).

Other types of macrophages are located at the CNS interface, such as meningeal macrophages, perivascular macrophages and choroid plexus macrophages (39–41). For decades these macrophages were believed to be derived from blood-borne monocytes, however new evidence suggests that at least some of them also arise from yolk sac precursors, being closer to microglia than to monocytes in their ontogeny (39). Nevertheless, microglia and these non-parenchymal macrophages remain distinct cell populations, with different locations, functions and morphologies (39).

### 2.2 Microglial functional diversity

Microglia possess a remarkable plasticity that allow them to perform a plethora of functions in the CNS during development, health and disease. As immune effectors of the CNS, microglia are well known to play crucial roles in response to injury and infection (42). Microglia express pattern recognition receptors (PRR) that allow them to recognize molecular patterns associated with pathogens and tissue damage (43). Upon PRR activation, microglia undergo a morphological- and physiological- transformation that leads to the release of pro-inflammatory cytokines, and the phagocytosis of pathogens and cell debris (44, 45). Conventional descriptors of ‘microglial activation’ include a change in functional behavior, as well as the migration to the site of injury, retraction of processes, and ‘compacting’ the cell body into an amoeboid morphology (23, 24, 46). Many of the microglial functions and morphological changes during the innate immune response, and in the diseased CNS, are extensively discussed in the literature (47–52).

In addition, microglia play crucial roles during the embryonic and postnatal development of the CNS. In rodents, microglia display an amoeboid morphology, typical of a phagocytic phenotype (53, 54). Accordingly, it has been shown that microglia control cortical neuron populations through phagocytosis of neural precursor cells (NPC) in rats and primates (55). Also, it has been described *in vivo* that microglia actively engage in phagocytosis of apoptotic cell bodies in the developing optic tectum and spinal cord of zebrafish (56, 57). In mice, *in utero* depletion of microglia during gestation, through CSF1R inhibition, led to the accumulation of dead cells in the hypothalamus and a significant increase in cell death throughout the developing hypothalamus, probably due to the lack of microglia-mediated elimination of apoptotic and dead cells (58). Interestingly, manipulating microglial activation had direct effects upon both the precursor cells population and the postnatal neuronal population. Microglial activation enhancement led to a decreased number of NPCs, while microglial deactivation increased the NPCs pool of cells (55). Microglia-mediated phagocytosis of synaptic material, known as synaptic pruning [reviewed in (59)], is crucial for the formation of neuronal pathways and the refining of neuronal circuits during development (60–63), ultimately affecting axonal growth, neuronal positioning and CNS cytoarchitecture (64).

Microglia have also been described to play key roles in the hypothalamus, were they influence the circuitry and signaling of the melanocortin system, responsible for the control of body weight and food intake (58, 65, 66). Administering PLX5622, a CSF1R-inhibitor, to pregnant mice achieved the *in utero* depletion of embryonic microglia (58), having postnatal effects upon energy balance. At postnatal day 4 (P4), pups from females treated with PLX5622 during pregnancy showed a reduction of 45% in the number of POMC neurons (58), neurons involved in the hypothalamic regulation of food intake through the release of anorexigenic peptides (67). The reduction of POMC neurons in PLX5622-exposed pups was accompanied by a significant increase in body weight gain from P5 to P15, when compared to pups from control females (58). These results show that depletion of microglia during gestation can affect the development of hypothalamic satiety circuits and have lasting effects upon body weight gain after birth (58). Embryonic microglia in the hypothalamus have also been shown to respond to different stimuli during gestation (68–71). *In utero* electroporation (IUE) is a procedure used to introduce plasmid DNA into the murine embryonic CNS (72). It has been shown that IUE induces morphological and gene expression changes in hypothalamic microglia, concomitant with an increased cell death in the developing hypothalamus (68). Furthermore, embryonic microglia interaction with radial glial cells (RGC) in the hypothalamus after IUE led to microglia-mediated degeneration and phagocytosis of RGC (69). Further evidence of the role of embryonic microglia during gestation has been shown in response to maternal stressors in a gestational cold stress model (70). In this study, cold exposure of pregnant mice led to an increase in the secretion of CCL3 and CCL4 by a subset of hypothalamic microglia, adjacent to neural stem cells (NSC), in the pups (70). Interestingly, this effect was only seen in male pups and was coincident with a decrease in the number of oxytocin neurons in the paraventricular nucleus of the hypothalamus (70). This effect seems to be CCL3 and CCL4-dependent, since these molecules also affected the proliferation and differentiation of hypothalamic NSC *in vitro* (70). Embryonic microglia have also been shown to be affected by gestational exposure to bisphenol A (BPA) (71). Exposing pregnant mice to BPA led to an increase of microglia numbers in the hypothalamus and changes in the morphology of microglial cells of the pups, showing higher ramification and higher number of phagocytic cups (71). These studies highlight the responsiveness of embryonic microglia to environmental factors, with lasting effects into the postnatal stages. The importance of hypothalamic microglia upon energy balance has also been shown in adults, where the microglia-specific disruption of leptin signaling caused hyperphagia and accelerated body weight gain, with concomitant loss of POMC neurons (66).

The microglial role during development goes beyond its phagocytic capacity, being able to secrete neurotrophic factors to promote neuronal survival, neurogenesis and oligodendrogenesis during early postnatal development (73, 74). In mice, it has been shown that microglia directly contribute to the survival of layer V cortical neurons through the secretion of the trophic factor insulin-like growth factor 1 (IGF-1) during postnatal development (74). In rats, microglia enhanced neurogenesis and oligodendrogenesis in the subventricular zone through the secretion of pro- inflammatory cytokines such as IL-1*β*, IL-6, TNF-*α* and IFN-*γ* (73). These studies highlight the importance of microglia during different stages of CNS development and maturation, not only being involved in the differentiation of other cell types but also refining the neuronal circuitry and CNS organization.

Microglial effects upon neuronal connectivity are also not limited to the developmental stage. In the adult CNS, microglia have been shown to engulf synaptic material in both the healthy brain and in neurodegenerative conditions (13, 75–77). Early ultrastructural studies described microglia-mediated displacement of synaptic terminals, suggesting that microglia actively participate in synaptic plasticity (78–80). More recently, microglia have been related to the elimination of synapses during adulthood and ageing across different regions of the CNS (13, 14, 81, 82). These functions suggest a key role of microglia in the modifications to the neuronal network in response to stress (81), memory maintenance (77), or in experience-dependent behavioral adaptation through synaptic plasticity (13). Interestingly, microglial ablation in adult mice led to a robust increase of the synaptic density in the hippocampus (83). Furthermore, it has been shown that blocking microglial BDNF (brain-derived neurotrophic factor) secretion, leads to a reduction in the formation of cortical dendritic spines associated with deficits in learning. This strongly suggests that microglia are not only involved in synaptic eliminations but also in synaptogenesis, with direct implications for learning-dependent plasticity (84). Altogether, this data show that microglia are involved in synaptic remodeling *via* both synaptogenesis and elimination, directly influencing neural plasticity.

## 3 Morphophysiological traits of microglia

### 3.1 The classic (and outdated) two-state paradigm

The morphological plasticity of microglial cells was already acknowledged by Río-Hortega in his 1919 series of papers about microglia [reviewed in (1)]. He described the morphological changes experienced by microglia after neuronal insult as: *“The first phenomenon observed in microglia that evidences their distress in brain pathological processes is an increase in volume which mainly affects their dendrites [“processes”]. Hypertrophy [“increased cell size”] of microglial cell bodies is observed in some cases, and a more or less active hyperplasia [“increased cell density”] can also be observed”* (1). Río-Hortega also referred to the gain of migratory and phagocytic characteristics in neurodegenerative processes: *“The nomadic character of microglia is best observed in neurodegenerative processes, during which the apparent rest that they enjoyed in the normal state turns into migratory and phagocytic activity”* (1). This led to the assumption that microglia exist in two different states: i) ‘Resting’, characterized by a highly ramified morphology and limited phagocytic and migratory activity, and ii) ‘Activated’, characterized by amoeboid shape, high motility, with phagocytic and proliferative capacities. For most of the last century this ‘two-state paradigm’ has been widely adopted, contributing to the misconception that microglia in the healthy brain were functionally quiescent or dormant (85, 86). Equally, the ramified-to-amoeboid transition observed in microglia during infection, trauma or pathological processes was inferred as the key criteria for microglial activation (87). Morphometric parameters such as sphericity, volume, cell body area, number of processes, length of processes, number of endpoints, number of nodes or microglial process area have been extensively used in the assessment of microglial morphology (56, 88, 89). Often these parameters are used to measure how ramified or amoeboid a microglial cell presents itself, using these as an indication of its activation state.

It is now well accepted that microglia undergo a morphological and functional transformation upon neuronal insult. Microglia do indeed migrate to the site of lesion, perform their phagocytic capacity to clear debris or eliminate pathogens and adopt an amoeboid morphology (22, 90). However, it has become evident that microglia are not dormant or quiescent in the so-called ‘resting’ state. *In vivo* studies have demonstrated that ramified microglia are quite dynamic, and their processes are continuously moving to survey the CNS parenchyma (91, 92). It has been convincingly demonstrated that microglia are very active in the healthy brain, beyond their direct immunological response patterns, playing important roles in synaptic plasticity, neurotrophic support, myelin remodeling, and maintaining homeostasis in the CNS (2, 4, 7). Hence, the morphological changes that were considered for many years as the key indicator of microglial ‘activation’ rather correspond to a morphophysiological transition that reflects a change in function. Further indication that the two-state paradigm fails to accurately reflect the spectrum of microglial phenotypes is the introduction of diverse categories of microglia functions in the literature, including surveillant microglia, proliferating microglia, pruning/neuromodulatory microglia, phagocytic microglia, and inflammatory microglia (93).

### 3.2 Factors determining the morphophysiological diversity of microglia

Microglia have been historically considered a homogenous population of cells. However, the regional, functional and morphological diversity of neurons, as well as the highly specialized organization of the CNS, suggest that microglial characteristics might reflect a similar heterogeneity (16, 88). Over the last 20 years, it has become more and more apparent that microglia show region-specific characteristics that are also affected by age and sex in the developing and adult CNS, as discussed below.

#### 3.2.1 Region

Early studies in adult mice showed variations in cell density throughout the brain that were accompanied by diverse morphologies of microglia depending on brain regions; with radially orientated arborized cells found abundantly throughout the grey matter, longitudinally branched elongated cells in the white matter, and compact amoeboid cells found around the circumventricular organs (16). Recent studies have also demonstrated region-specific differences in lysosome content and membrane properties of microglia throughout the brain (94, 95). Transcriptomic profiling of different peripheral macrophage populations also demonstrated inter-region variability (96). Further studies using RNA sequencing of microglia have also highlighted strong regional differences. For instance, it has been shown that the expression of genes related to the phagocytic capacity of microglia differ between different regions in mice. Using microglia-specific mRNA extraction, Ayata and colleagues showed that expression of cell-clearance genes was significantly more prominent in cerebellar microglia when compared to striatal or cortical cells (97). The fact that the cerebellum shows higher levels of neuronal loss compared to the striatum or cortex, suggests that microglial phenotypes may be greatly determined by the surrounding microenvironment. Another study in mice used single-cell RNA sequencing (scRNA-seq) to investigate the regional differences of microglia at different developmental stages (6). Interestingly, Li and colleagues showed that early postnatal microglia located in highly proliferative regions showed a similar gene signature to that of degenerative disease-associated microglia. Furthermore, the authors found that the transcriptome of microglia expressing homeostatic genes in the adult mice was very similar regardless of the brain region, suggesting that the regional differences may change among different developmental stages, and that age is also a factor relevant for microglial phenotypic differences (6).

#### 3.2.2 Age

Microglia migrate to the CNS at the very early stages of embryonic development and remain resident in the CNS parenchyma with self-renewal capacity throughout life. Hence, microglia are present during the developmental, adult, and aging stages of the CNS, presumably playing important roles by adapting to the different processes at different stages. The intense phagocytic activity observed during development correlates with the typical amoeboid morphology of phagocytic microglia (53, 54). On the other hand, microglia in the adult CNS show ramified morphology that is often considered an indication of microglial maturation (12, 98). Nonetheless, this loss of amoeboid morphology in adult microglia does not necessarily correlate with a loss of phagocytic activity, as many studies have described that microglia are phagocytically active even in the ramified state (11, 62). Furthermore, a recent study investigating microglial heterogeneity in mice at different ages using scRNA-seq showed that microglia display a higher transcriptomic diversity in the developing, aged, and diseased brain, compared to the adult microglial population (5).

#### 3.2.3 Sex

The CNS presents a series of sex-specific characteristics regarding its anatomy, physiology, morphology, and epigenome (99–101). Sex-specific differences in the density, morphology and phagocytic activity have been described during development and in the adult mice (102), probably due to differential hormonal surges throughout life. This sex dimorphism not only comprises neuronal traits but also entails differences in non-neuronal cells. One of the first studies describing sex-specific differences in the microglial population reported that female mice showed an increase in microglia number, of at least 30% in the hippocampus compared to male littermates at three different ages (103). Another study highlighted that microglia number and morphology are affected by sex, age, and brain region (53). Male rats showed more deramification of microglia, the retraction of microglial processes and microglia adopting an amoeboid shape at early postnatal stages while females showed an increased number of deramified cells in juvenile and adult individuals. These differences in microglial number and morphology were also accompanied by differences in the gene expression of cytokines. These data suggest that microglial morphology, and likely their physiological state, are modulated by sex at different ages and regions. Subsequent studies suggested that microglia might have a relevant role in repressing the feminization of the brain, a process characterized by changes that sexual receptivity and maternal behavior (104). Specifically, one study described an increase in the number of microglia present in the preoptic area (POA) of neonatal male rats, showing a significant role of microglia in the synaptic patterning of the POA, crucial for masculinization of the brain and behavior (105). Interestingly, they also reported differences in the morphology of microglial cells in the POA, where neonatal males had twice as many amoeboid microglia compared to female littermates.

Some studies have also reported sex-specific traits in pain modulation directly related to microglia. For example, male mice were reported to show a TLR4-dependent activation of microglia during inflammation and neuropathic hypersensitivity (106). Another study assessing sciatic nerve hypersensitivity found that microglia were responsible for mediating hypersensitivity in male mice, while the response in females was mediated *via* T-cells (107). This difference was deemed mainly due to hormonal differences, as castrated males lacking testosterone lost the microglial response, while hypersensitivity was blocked by targeting microglia in females lacking T-cells and treated with testosterone (107). Differences in microglia-mediated pain hypersensitivity were also reported in rats, where inhibition of the P2X4R pathway in microglia resulted in the elimination of hypersensitivity in male rats but not in females, despite both displaying reactive microgliosis (108). Pharmacological modulation of neuropathic pain was also achieved with metformin, an antidiabetic drug, eliminating pain and microglial ‘activation’, determined by the activation marker Iba-1, in the spinal cord of male but not female mice (109). Dimorphic effects of morphine between female and male rats might also be related to sex-specific microglia differences. It has been reported that the increased number of deramified microglia in the periaqueductal gray is responsible for the attenuated effects of morphine seen in female rats (110). Interestingly, this was reversed through microglial modulation by blocking TLR4 in females. Altogether, these studies demonstrate that microglia might play a key role in pain modulation and that this is greatly influenced by sex-specific differences.

### 3.3 Microglia morphophysiological diversity

**Figure 2:**
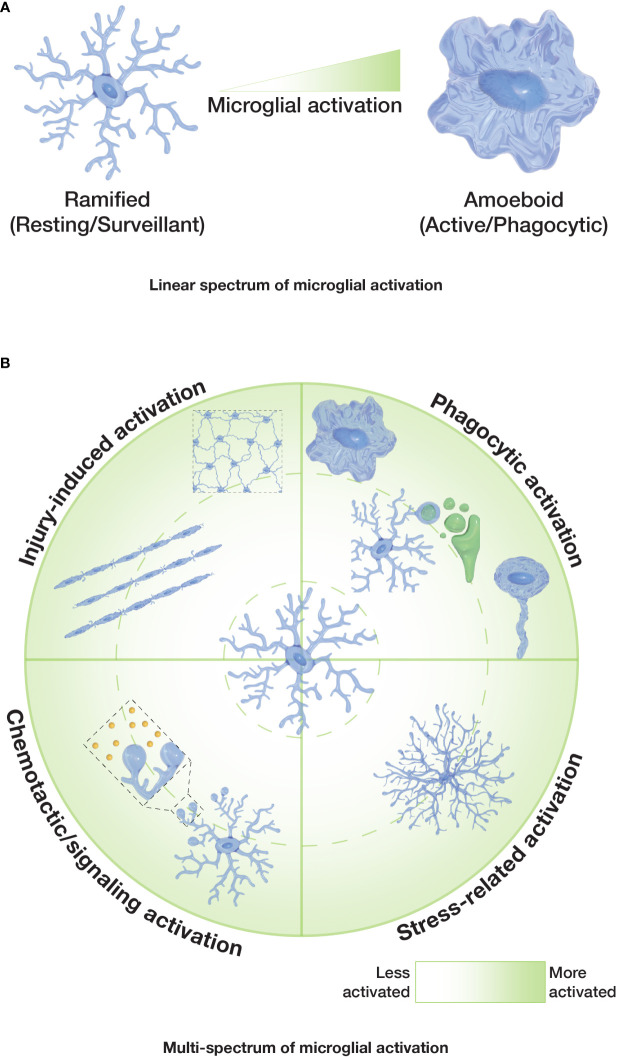
Spectrums of microglial activation. **(A)** Classical or ‘linear spectrum’ of microglial activation where ramified microglia are considered non-activated/resting, and amoeboid microglia are considered fully activated. Other microglial morphologies would reflect an intermediate state. **(B)** Proposed ‘multi-spectrum’ that reflects changes in microglial morphologies and function in a radial pattern, with context-dependent microglial activation.

Although most studies use the ramified-amoeboid spectrum (**Figure 2**) to classify the morphology of microglial cells and generally directly correlate the phenotypic appearance with the physiological response, several studies have identified alternative morphologies (**Table 1**). Importantly, these studies did not find a clear correlation between increased expression of pro- or anti-inflammatory markers with such morphologies. This suggests that microglia are capable of modifying and adapting their morphology in response to stimuli unrelated to their activation or immunological status.

**Table 1:** Microglial morphologies and their reported functions.

Morphology	Functional characteristics	Model	References
**Ramified**	Classically considered a ‘resting’ state.	Human, mouse, rat, zebrafish	(7, 13, 57, 88, 91, 92, 111, 112)
	Frequent extension/contraction of microglial processes.		
	Surveillance of CNS parenchyma and neuronal activity.		
	Neuroprotective role during excitotoxicity.		
**Amoeboid**	Classically considered an ‘activated’ state.	Human, mouse, rat, zebrafish	(16, 23, 24, 46, 53, 56, 57, 59, 113)
	Transformation to amoeboid morphology in response to infection, injury and/or pathological processes.		
	Phagocytically active.		
	Key roles in the healthy brain, eliminating debris and apoptotic cells.		
**Bulbous endings of microglial processes**	Transient formation of bulbous structures in the apex of microglial processes.	Mouse, zebrafish	(91, 114–116)
	Involved in the chemotactic response to neuron-released ATP gradients.		
	Also involved in monitorization and control of neuronal activity.		
**Ball-and-chain structures**	Present in immunologically unchallenged microglia.	Mouse, rat, macaque, zebrafish	(11, 55, 117–121)
	Phagocytically active.		
	Involved in the phagocytosis of apoptotic cells, neural precursors and myelin sheaths.		
**Hyper-ramified**	Hyper-ramification of processes in response to acute and chronic stress.	Mouse, rat	(122–125)
	Possible role in stress-related synaptic modifications.		
**Honeycomb**	Formed in response to BBB leakage after compression-TBI.	Mouse	(126)
	Several microglia cells retract most processes, except 3-4 to form a contiguous network.		
**Jellyfish**	Formed in response to astrocytic death after compression-TBI.	Mouse	(126)
	Microglia extends a single non branching process.		
	Phagocytically active.		
**Rod microglia**	Elongated and narrowed soma.	Human, mouse, rat	(127–131)
	Thin polar processes.		
	Can form multicellular ‘trains’ of several rod microglia.		
	Rod microglia align adjacent to injured neurons.		
	Described in several pathological processes.		

#### 3.3.1 Microglia fulfill important roles in their ramified state

Deramification (the transition to an amoeboid state) has been considered an indication of microglial activation for many decades. However, many studies have shown that microglia in the so-called ‘resting’ state, characterized by a highly ramified morphology (**Figure 1A**), are actually extremely dynamic and active. Early evidence was provided in studies using microglia-reporter mice and two-photon microscopy, allowing the observation of microglia and their processes *in vivo* (91, 92). Those studies revealed that while the soma remained relatively static with few signs of migratory behavior; microglial processes were in constant motion, establishing transient contacts with neurons, astrocytes and blood vessels (92). This constant and rapid extension-contraction of the microglial processes is thought to be part of a continuous surveillance of the CNS microenvironment, allowing the CNS parenchyma to be monitored every few hours (7, 92). Surface receptors allow microglia to detect changes in the environment (82, 132) and to monitor neuronal activity through transient contacts with synaptic structures (7, 13). As we discuss below, the role of ramified microglia is not limited to surveillance. It has been shown that microglia can present with bulbous processes, forming ball-and-chain structures (**Figure 1C**), which are believed to be an indicator of phagocytosis of small amounts of debris or tissue without transitioning to an amoeboid shape (11). This suggests that such microglial phagocytosis of synapses and apoptotic material might not require the loss of ramified morphology, contrasting with observations in pathological conditions, where phagocytosis is generally performed by amoeboid microglia (59). Some studies have further described ramified or hyper-ramified microglial phenotypes (**Figure 1D**) that are actively engaged in other scenarios, such as the physiological response to stress and excitotoxicity. For instance, chronic stress resulted in a higher expression of Iba-1 and the hyper-ramification of microglia in the prefrontal cortex of rats, correlating with an enhanced activation of neurons in that region and impaired spatial working memory (122). Another study used mouse organotypic hippocampal slice cultures and showed that ramified microglia exert neuroprotective effects upon neurons during NMDA-induced excitotoxicity (111). Vinet and colleagues demonstrated that some neurons in the CA3 and DG regions of the hippocampus were resistant to excitotoxicity. This resistance was lost after ablation of ramified microglia, severely affecting the viability of neurons, while replenishment of microglia-free slices restored the resistance in those regions (111). Interestingly, the results in this study suggest not only that ramified microglia are able to exert neuroprotective roles in pathologic processes, but also that this function might be region-specific as other hippocampal regions showed different responses.

#### 3.3.2 Alternative microglial morphologies and their function

##### 3.3.2.1 Bulbous endings of microglial processes

As early as 2005, in one of the first *in vivo* studies describing the dynamic nature of microglial processes, the authors described that microglia extended their processes in response to local brain injury in mice (91). Interestingly, this response was mimicked by local ATP injections and characterized by bulbous endings of microglial processes (**Figure 1E**). These observations were confirmed in later studies where the outgrowth of microglial processes was associated with a chemotactic response to gradients of neuron-released ATP, through NMDA receptors on the surface of microglial processes (114, 115). Both studies reported that the formation of these bulbous endings was transient and was reversed when ATP application was terminated. The role of ATP in inducing the formation of bulbous structures in microglial processes was also described *in vivo* in zebrafish (116). The authors revealed that these bulbous microglia-neuron interactions were dependent on neuronal Pannexin-1 hemichannel, permeable to ATP, and ATP/P2 receptors in microglia. Interestingly, they showed that microglia were more prone to form bulbous contacts with neurons depending on their activity, as microglial processes preferentially moved towards neurons showing higher frequencies of Ca2+ activity. Most importantly, they showed that ramified microglia were able to downregulate the activity of those neurons contacted with such bulbous structures. These results suggest that ATP plays an essential role in signaling between neurons and microglia, that microglia are capable of sensing neuronal activity, and that this activity can be modulated *via* microglial bulbous processes.

##### 3.3.2.2 Ball-and-chain structures

After the initial *in vivo* observation that ‘resting’ microglia actively and constantly modify the length of their processes to monitor the CNS parenchyma (91, 92), another study showed in the adult murine CNS that immunologically unchallenged ramified microglia are able to phagocytose apoptotic cells (11). The authors described a novel phenomenon in hippocampal sections of 1 month-old mice, where some microglial processes adopted a so-called ball-and-chain structure, in which a spherical phagocytic pouch (ball) was formed at the tip of a microglial terminal branch (chain) during apoptotic clearance (**Figure 1C**). Other studies also described this morphological type of interaction during early development. In the postnatal (P13) mouse subventricular zone (SVZ), microglia engulfed apoptotic dividing cells by forming phagocytic cups at the tip of the processes (117). In macaque, microglia phagocytosed neural precursor cells in the developing neocortex (E80), through enveloping the cell with a phagocytic structure formed in the distal portion of one of its processes, forming a ball-and-chain structure (55). More recently, a study in rats described that ball-and-chain structures were prominent in early post-developmental SVZ (P10) (118). Interestingly, these structures were also abundant in later stages (P40) in rats exposed to neonatal hypoxia-ischemia surgery, suggesting that they might also be implicated in the response to long-lasting effects of perinatal neuronal challenges. Ball-and-chain structures were also observed in ramified microglia in the developing cerebellar cortex of rats (119). Even though the authors do not explicitly use the ball-and-chain terminology, a recent *in vivo* study found that microglia phagocytose myelin sheaths during the development of the optic tectum and spinal cord of juvenile zebrafish, in a fashion that resembles the ball-and-chain structure (120). They reported that phagocytic events, identified through calcium signaling, led to the phagocytosis of portions of myelin sheaths. Interestingly their images show elongated microglia with short processes that in some cases showed a phagocytic pouch at their tips, resembling the ball-and-chain morphology. This process was regulated by neuronal activity, with microglia engulfing more myelin when neuronal activity was reduced or suppressed (120). Microglial phagocytosis through ball-and-chain structures has also been shown to be involved in the masculinization of social behavior in juvenile rats (121). Microglia in the developing amygdala of male rats have been shown to have an increased phagocytic activity compared to female juveniles during the first postnatal week (121). The microglia formed ball-and-chain structures that consequently engulfed and phagocytose newborn astrocytes, a process that was testosterone-induced and dependent on endocannabinoid signaling (121). Moreover, phagocytosis of the newborn astrocytes by ball-and-chain structures was complement-dependent, since blocking complement receptor 3 (CR3) signaling increased astrocytic survival and prevented social masculinization (121).

##### 3.3.2.3 Hyper-ramified microglia

Ramified morphology of microglia (**Figure 1A**) has been considered for many decades as an indication of quiescence or a ‘resting’ state. However, recent studies in rodents have described a process of microglial hyper-ramification (**Figure 1D**) in response to acute and chronic stress. A study in rats described a non-injury-related hyper-ramification of microglia in the medial prefrontal cortex in response to chronic stress (122, 123). The authors reported that after chronic restraint stress, microglia presented higher ramification through an increase in the branching points of its processes. This hyper-ramification was accompanied by an upregulation of *β*1integrin, a protein that has been implicated in promoting microglial ramification. This effect was rescued after the administration of minocycline, a microglial inhibitor. Another study, using the chronic despair model (CDM) to induce stress-related depressive-like behavior in mice, observed a change in microglial morphology characterized by longer processes and increased branching in wild-type mice (124). Interestingly, CX3CR1-deficient mice showed enhanced resistance to the effects of CDM in regard to microglial morphology, and the administration of the anti-depressant venlafaxine reversed the hyper-ramification of microglia in the wild-type mice. These results suggest that the fractalkine-CX3CR1 axis might be involved in the neuron-microglia signaling during stress-induced depression. Furthermore, the authors reported that the administration of venlafaxine increased the expression of synaptic plasticity marker Arc/Arg 3.1 in wild-type mice but not in CX3CR1-deficient mice, suggesting that potential synaptic modifications are happening in response to stress. A recent study using a mouse model of post-traumatic stress disorder (PTSD) reported long-lasting fear response, decreased locomotor activity, and impaired behavior accompanied by an increased number of hyper-ramified microglia and loss of dendritic spines in a region-specific manner (125). Overall, these results suggest that microglial hyper-ramification can be a stress/depression-specific response that might also be implicated in synaptic modifications.

##### 3.3.2.4 Honeycomb and jellyfish microglia

Other microglia morphologies have been reported in response to neuronal injury and glia limitans rupture after traumatic brain injury (TBI) in mice (126). The glia limitans is a layer of astrocytes endfeet processes that separate the CNS parenchyma and the perivascular space, just beneath the BBB (133). After thinning of the murine skull, a model of compression-mediated TBI, the authors reported leakage from the BBB through to the CNS parenchyma, mainly due to astrocytic death in the glia limitans (126) In response to this phenomenon, microglia retracted most of their processes except for two or three, forming a contiguous and highly connected network resembling a honeycomb structure (**Figure 1F**). Interestingly, this seemed to be a coordinated response of several microglial cells that surrounded surviving astrocytes to prevent further disruption of the glia limitans. The same study also reported a morphological transformation of honeycomb microglia into a shape that resembled a jellyfish, by the extension of a single, non-branching process (126) (**Figure 1G**). This transition from honeycomb to jellyfish-shaped microglia was observed in response to astrocytic death, likely suggesting the formation of a phagocytic cup. These structures were directly related to ATP-mediated microglial activation through the P2RY12 pathway. When the astrocytic release of ATP was inhibited with carbenoxolone, honeycomb and jellyfish structures were inhibited, and microglia remained ramified. These findings may suggest that honeycomb and jellyfish microglia are contributing to maintenance of the BBB integrity after TBI. A role of microglia in BBB integrity after brain injury was also demonstrated in mice, where photoablation of microglia and the inhibition of P2RY12 both resulted in the impairment of BBB closure (134). Inhibition of microglia through P2RY6 antagonism likewise resulted in increased parenchymal cell death 12 hours after compression injury (126).

##### 3.3.2.5 Rod microglia

Until recent years, ramified and amoeboid morphologies have been the predominant morphological descriptors of microglia. However, already in 1899 Franz Nissl described cells with rod-like shape (“Stäbchenzellen”) in the post-mortem brain of patients with general paresis of the insane. These cells are now recognized to be a differentiated morphology of ‘activated’ microglia, commonly referred to as rod microglia (127, 128, 135) (**Figure 1H**). Later studies from Ramón y Cajal, Río-Hortega, Achúcarro and Alzheimer helped to establish rod microglia as a neuropathological marker of general paresis, cerebral atrophy and multiple sclerosis (128, 135). Further post-mortem studies during the first half of the 20th century showed rod microglia in the cortex of patients with malaria, Alzheimer’s disease, multiple sclerosis, epilepsy, and encephalitis (135). More recent studies defined rod microglia as cells with elongated and narrowed soma, with polarized thin processes mainly in the apical and basal ends of the cell due to the retraction of planar processes (128, 129). The authors observed the morphological transition of microglia adopting a rod-like shape after diffuse brain injury in rats, starting at 1-day post-injury and being prominent at 7 days post-injury (128). Similar results were observed after optic nerve transection in rats, where the presence of rod microglia was prevalent in the retina starting at 7 days after injury and becoming more evident between 14 and 21 days after the nerve transection, with more than 80% of the retinal microglia displaying a rod-like shape (130).

A study of human post-mortem brain samples revealed a correlation between age and the presence of rod microglia in the hippocampus and cerebral cortex (131), suggesting age as a possible factor. The authors also found an increased number of rod microglia in the parietal cortex of samples from patients with Alzheimer’s disease. Interestingly, no correlation was found between the history of traumatic brain injury and the presence of rod microglia, suggesting that the appearance of rod microglia after an acute brain injury might resolve after an undetermined period of time (131). Rod microglia have been usually described to adopt specific orientations and being adjacent to dendrites and axons of injured neurons (22, 128–130), suggesting a possible neuroprotective role by creating a barrier to protect uninjured neurons (129). Furthermore, it has been described that rod microglia align to each other after brain injury and optic nerve transection, forming structures resembling trains of rod microglia (**Figure 1H**) (128–130), suggesting a coordinated response of multiple rod microglial cells.

## 4 Discussion

It is remarkable that Pío del Río-Hortega’s conclusions about the dynamic nature of microglia are still valid today considering his observations were based on static examinations of the CNS (1). In 1919, Río-Hortega already mentioned the mesodermal origin of microglia and the correlation existing between morphology and microglial function, noting the ability of these cells to change their shape in response to different stimuli. However, being the only phagocyte resident in the CNS parenchyma, microglia were almost exclusively labeled to be responsible for the initiation and resolution of ‘neuroinflammatory’ reactions. Thus, it is not surprising that other roles of microglia, especially in the healthy CNS, were overlooked. Microglial dynamics have generated significant interest in the field of neurological research, arguably due to technological advances that, for example, allowed *in vivo* visualizations of ‘resting’ microglia under minimally invasive condition (91, 92). Under near-physiological conditions, these *in vivo* observations transformed the field by demonstrating the dynamic nature of unchallenged microglia that were considered dormant or quiescent for many years. These observations helped to understand that microglia are not only active in response to pathological changes in the CNS when they adopt an amoeboid shape and increase their phagocytic activity, but that ramified microglia also play very important roles in maintaining CNS homeostasis without losing their ramified morphology.

The use of post-mortem human tissue from brain donors has been especially useful in studying microglial function in neuropathology, including different psychiatric and neurodegenerative disorders (93, 136–139). Post-mortem studies provide a unique opportunity to correlate a characteristic microglial morphology to different disease stages, neuropathological hallmarks and other clinical parameters such as disease severity and duration (140). However, variations of clinical and autopsy-related parameters between cases can impact microglial morphology for histological studies, potentially complicating some interpretation of the results. Also, studying the implications of microglia in non-diseased brains and during the onset and early stages of diseases has been difficult, mainly due to the scarce availability of such tissue (140). Post-mortem studies in animal models have likewise contributed to our understanding of processes such as microgliosis, the proliferation of reactive microglia in response to acute CNS injury and aging (141), or microglia-mediated synaptic modifications (78, 87). For instance, studies using rats undergoing facial nerve axotomy were crucial to reveal the role of microglia in synaptic plasticity through a process called synaptic stripping (78, 113), where microglia have been shown to selectively displace synapses from injured neurons (142). This led to a renewed interest in microglial function, which has since become a highly debated topic in glial biology and neuroscience because of its important implications in CNS homeostasis, disease outcomes, and even potential therapeutic interventions (1).

*In vivo* studies of microglial responses have revealed unprecedented insights into the behavior, physiology, and spectrum of these cells. It has become apparent that such visualization models can be instrumental in deciphering the diverse spectrum of microglia phenotypes and physiology. In recent years, many studies have investigated the dynamic nature of microglial physiology in both the healthy and diseased brain, from the early stages of embryonic development throughout the adult brain and aging, using *in vivo* and *ex vivo* animal models (111, 143–145). These approaches have, for instance, clarified the ontogeny of microglia in mice (26) and zebrafish (29), revealed the role of microglia in eliminating apoptotic cells in zebrafish during development (146) and in the developed CNS (56), identified microglia-mediated synaptic modifications in mice (7, 147), or the microglial proliferation in animal models of disease and aging (34, 148). More recently, the appearance of new molecular techniques, and in particular single-cell RNA sequencing, has made it possible to define new roles and new subpopulations of microglia, such as a recovery-related subpopulation in a mouse model of nerve injury (149), and microglial subpopulations expressing different transcriptional profiles in mice depending on the region, age, or disease state (5).

Many of these findings have illustrated various forms of interactions between microglia and other cells, interactions that differ from the classic ramified/amoeboid paradigm. These studies not only describe unique microglial morphologies, but also demonstrate that the classical associations (‘ramified’ equating ‘resting’ microglia; ‘amoeboid’ equating ‘active’ microglia) are not appropriate in many cases (**Figure 1**). One of the first *in vivo* studies of microglia in mice already described the formation of a unique type of bulbous structures at the tips of microglial processes (**Figure 1E**) in response to neuronal damage and injections of ATP (91). This was later confirmed by other studies in mice (114, 115) and zebrafish (116). These structures appear to be closely related to the chemotactic attraction of microglia by ATP and, as observed in zebrafish, might be also involved in the regulation of neuronal activity (116). Other studies have also highlighted that branched microglia can be phagocytically active. One of the most observed microglia morphologies under physiological conditions is the ball-and-chain structure (**Figure 1C**), first described *ex vivo* in adult mice hippocampal sections (11). These structures have also been observed in the developing hippocampus of mice (117), the developing cerebral cortex of macaques (55), in the developing cerebellum in the rat (119), after neonatal hypoxia-ischemia also in rats (118), and remodeling myelin sheaths in zebrafish (120). Several features of these ball-and-chain structures remain unknown, for example whether this phagocytic structure arises exclusively when microglia are immunologically ‘inactive’ (i.e., in the absence of pathogens in the CNS or BBB disruption), or whether the volume of the phagocytosed material is a contributing factor. Another example where branched microglia are phagocytically active is the process of trogocytosis, where tiny pouches form on the surface of microglial processes to eliminate presynaptic structures in mice (147). The retraction of microglial processes has often been considered as indicative of microglial activation. However, some studies have described that microglia can adopt a hyper-ramified morphology in response to stress (122, 123). Other studies of murine models of chronic stress and PTSD correlated microglial hyper-ramification with long-lasting behavioral and motor impairment accompanied by synaptic modifications (124, 125).

Some studies have not only observed unique microglial morphologies but have also described that microglia can form multicellular networks with a very well-defined structure. Such is the case for the trains of rod-shaped microglia (**Figure 1H**) formed in close relationship with neuronal structures after diffuse brain injury in rats (128), or the arrangement of microglial cells forming a structure reminiscent of a honeycomb (**Figure 1F**) after TBI in mice (126). This suggests that microglia can orchestrate a joint and coordinated response to CNS insults. It will be interesting to see future studies further unravel our understanding of inter-glia and inter-cellular communication in the CNS, such as the microglia-mediated activation of astrocytes and neuronal degeneration (150). Identifying the molecules and signaling pathways that trigger such inter-cellular responses or intra-cellular morphophysiological transformations will undoubtedly help us to understand the cues leading to microglial differentiation (morphologically and functionally).

Thus, it seems clear that the extraordinary heterogeneity and diversity of microglial cells requires the reconsideration of the outdated dogma of their different activation states being linked to one particular morphology. The classical paradigm of microglia depicts their morphology and activation as correlated and on a linear spectrum — with ramified microglia being non-activated on one end and the amoeboid morphology reflecting activated microglia on the other end (**Figure 2A**). This paradigm does not account for the great variety of microglia morphologies seen in the CNS and does not encompass the emerging evidence of much more diverse morphophysiological correlations. Certainly, microglial responses are extremely complex and diverse, making it difficult to establish exact correlations between microglial functions and specific morphologies. It is therefore important for researchers to assess microglial dynamics, taking into account the spatiotemporal changes of both microglial morphology and function.

Species-specific traits of microglia are also important when accounting for variation between microglia morphophysiologies. Despite sharing a highly conserved transcriptome, several studies have already highlighted gene expression differences between human and murine microglia (151–153). Another example is the expression of a surface lectin in microglia, Siglec-11, that seems to be unique to the human brain (154). Torres-Platas and colleagues performed a morphometric analysis of cortical microglia in both humans and mice (88). The most evident differences between mice and human microglia arise from the proportion of the different morphologies described and the shape of the cell bodies. Mouse cortex showed a higher proportion of ramified microglia (≥90%) with microglial somata being highly heterogeneous in their shape, while in human cortex ramified microglia represented 43% of all cells and the cell bodies were classified either as rounded or amoeboid (88). Specifically, ramified, amoeboid and rod morphologies have been described in multiple species, including humans and mice, and highlighted in this review (**Table 1**). Notably, these are considered classical microglial morphologies, all being described in the first half of the 20th century. It will be interesting to study human microglia by taking into account more recently described morphologies and avoiding the ramified-amoeboid linear spectrum, allowing a more accurate comparison between different species in the future.

For future studies, we suggest the usage of a continuous multi-spectrum (**Figure 2B**) for microglia morphologies and functions. Such a spectrum accounts for the variety of functional states for each microglia morphology and emphasizes their capacity to alter their ‘activation’ status, independently of their morphological pattern (e.g. it is not clear whether microglia can be ‘locked in’ in a morphological or functional state). Overall, it seems sensible for the field to develop context-specific ways to investigate microglial activation, beyond simply quantifying the ramification or sphericity of these cells. More standardized ways of describing the shapes that microglia can adopt and clearly associate them to particular functions will facilitate the comparison of different microglial responses in different settings and scenarios. *In vivo* studies of microglial dynamics may help to better define the morphological transitions of microglia and their interactions with other cells in the future.

## Author contributions

Conceptualization: AV-I and MM; data curation: AV-I and MM; writing—original draft preparation: AV-I, RR, CM, MM; writing—review and editing: all authors; visualization: AV-I and MM; supervision: AL, RC, ED, MG, and MM; funding acquisition: AL, CM, RC, MG, and MM. All authors have read and agreed to the final version of the manuscript.

## Funding

This work was supported by Australian Research Council (ARC) grants (DP150104472 and DP210103469), a Snow Foundation Fellowship (towards MM), and donations made towards MND research at Macquarie University.

## Acknowledgments

We wish to thank the Snow Foundation for their generous support towards establishing the transgenic zebrafish facility at Macquarie University and continued support of the researchers. We also wish to thank the zebrafish facility staff (past and present) for assistance in zebrafish care.

## Conflict of interest

The authors declare that the research was conducted in the absence of any commercial or financial relationships that could be construed as a potential conflict of interest.

## Publisher’s note

All claims expressed in this article are solely those of the authors and do not necessarily represent those of their affiliated organizations, or those of the publisher, the editors and the reviewers. Any product that may be evaluated in this article, or claim that may be made by its manufacturer, is not guaranteed or endorsed by the publisher.
